# Epidermal growth factor receptor variant III in head and neck squamous cell carcinoma is not relevant for targeted therapy and irradiation

**DOI:** 10.18632/oncotarget.15949

**Published:** 2017-03-06

**Authors:** Dominik Thomas Koch, Anja Pickhard, Lena Gebel, Anna Maria S. Buchberger, Murat Bas, Carolin Mogler, Rudolf Reiter, Guido Piontek, Markus Wirth

**Affiliations:** ^1^ Department of Otorhinolaryngology Head and Neck Surgery, Technical University of Munich, 81675 Munich, Germany; ^2^ Institute of Pathology, Technical University of Munich, 81675 Munich, Germany; ^3^ Department of Otolaryngology Head and Neck Surgery, Section of Phoniatrics and Pedaudiology, University of Ulm, 89070 Ulm, Germany

**Keywords:** head and neck squamous cell cancer, EGFR variant III, cetuximab, TKI, radiation

## Abstract

**Background:**

The epidermal growth factor receptor (EGFR) is an important regulator of cell growth and survival, and is highly variable in tumor cells. The most prevalent variation of the EGFR extracellular domain is the EGFR variant III (EGFRvIII). Some studies imply that EGFRvIII may be responsible for the poor response to the monoclonal EGFR-antibody Cetuximab, used therapeutically in head and neck squamous cell carcinoma (HNSCC). Due to inconsistent data in the literature regarding EGFRvIII prevalence and clinical relevance in HNSCC, especially its predictive value, we examined EGFRvIII-transfected cell lines and patient tissue samples.

**Results:**

In contrast to other recent publications, we were able to demonstrate EGFRvIII expression in HNSCC. However, we noted that the different detection methods yielded inconsistent results. Furthermore, our EGFRvIII transfected and EGFR wild type cell lines exhibited similar characteristics and response rates in the performed *in vitro* experiments.

**Materials and Methods:**

We conducted various inhibition and combined irradiation experiments using three EGFRvIII-transfected cell lines. Moreover, a patient cohort of 149 cases consisting of formalin fixed and paraffin embedded (FFPE) and fresh-frozen specimens was assayed via reverse transcriptase PCR (rtPCR) with gel electrophoresis and sequencing for EGFRvIII prevalence. In the rtPCR assays, we used five previously published EGFRvIII primers and EGFRvIII-positive glioblastoma tissue as a positive control. In addition, immunohistochemical staining was conducted.

**Conclusions:**

EGFRvIII can be detected in HNSCC patient samples. Nevertheless, the low prevalence and similar response rates to targeted drugs and irradiation *in vitro* cast doubt regarding the clinical relevance of EGFRvIII in HNSCC.

## INTRODUCTION

Head and neck squamous cell carcinomas (HNSCC) are the sixth most frequent tumor entity in the world [1]; thus, there is growing importance to gain an understanding of therapeutically relevant tumor characteristics. One striking characteristic of HNSCC is the high rate of epidermal growth factor receptor (EGFR) overexpression, as percentage rates of up to 90% have been documented [2].

The epidermal growth factor receptor (EGFR), is highly variable in tumor cells and an important regulator of cell growth and survival [3]. Thus, variations in EGFR have the potential to promote tumor growth and survival. Therefore, it seems natural to assume that these variations would influence EGFR-targeted therapies. Recently, several EGFR-targeting drugs have been developed to inhibit these EGFR-induced effects on tumor cells, including tyrosine kinase inhibitors and monoclonal antibodies.

In particular, the pharmacological therapy of HNSCC includes the monoclonal antibody Cetuximab which targets the extracellular domain of EGFR, suggesting high success rates due to the prevalence of EGFR overexpression. However, relatively few patients suffering from HNSCC benefit from Cetuximab [4], while all patients are subjected to serious side effects. To date, no clinically relevant predictor (e.g., K-Ras that can be routinely used in colorectal carcinoma [5]) has been identified. There is also no established correlation between EGFR overexpression and the benefits of Cetuximab therapy. Therefore, other predictive indicators need to be identified.

One mechanism of Cetuximab resistance could be the variation in the epidermal growth factor receptor. The most prevalent variation of the extracellular domain is the EGFR variant III (EGFRvIII), which is generated by the deletion of exons 2–7 in the wild type EGFR [6]. Thus, theoretically, Cetuximab cannot target the newly created and constitutively active EGFRvIII [7]. Several studies have implied that EGFRvIII could be responsible for Cetuximab resistance [8, 9]. However, other reports describe that EGFRvIII rarely occurs in HNSCC [10] or is not expressed at all [11].

To examine the relevance of the EGFRvIII in detail, we transfected HNSCC cell lines with the EGFRvIII receptor and conducted various inhibition and irradiation experiments. In addition, a patient cohort of 149 cases was assayed for EGFRvIII prevalence using reverse transcriptase PCR (rtPCR) and sequencing. Additionally, we conducted immunohistochemical staining for EGFRvIII. To clarify the inconsistent bibliographical data, we used five different primers in parallel for the EGFRvIII rtPCR assay.

## RESULTS

### HNSCC cell lines can be transfected stably with the EGFRvIII plasmid

**Figure 1:**
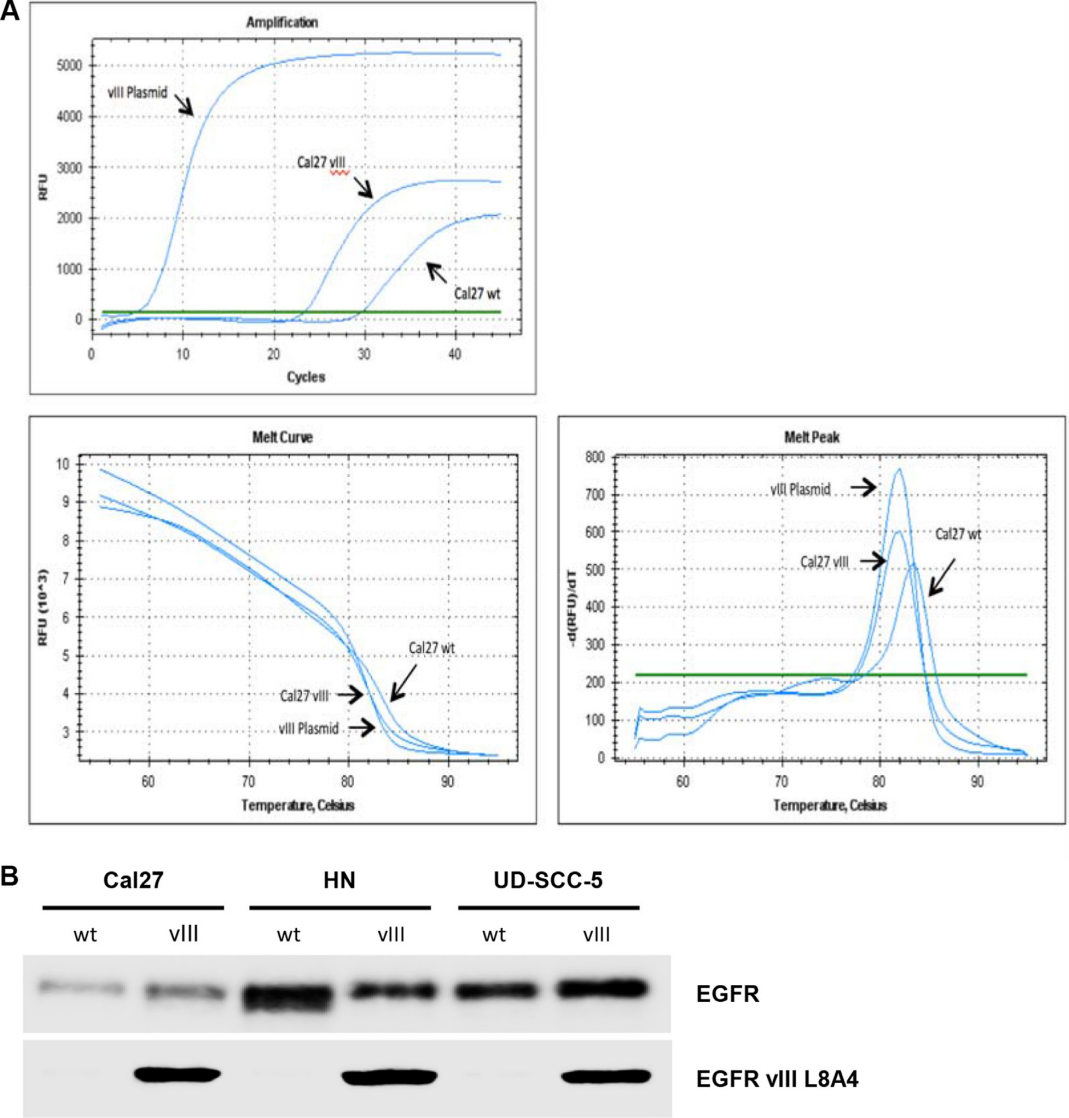
(**A**) EGFRvIII plasmid melting curve analysis of the transfected cell line, Cal27, and the wild type cell line, Cal27, demonstrating RNA expression of EGFRvIII. (**B**) In Western blot analyses we demonstrated the protein expression of the EGFRvIII variant in the transfected cell lines Cal27, HN, and UD-SCC-5.

We transfected three HNSCC cell lines with an EGFRvIII plasmid. Using rtPCR and western blotting, the efficiency of EGFRvIII transfection was determined (relevant pictures are displayed in the supplemental section). Figure 1A presents real time PCR analysis demonstrating stable transfection. Figure 1B shows the protein expression of the EGFRvIII variant in the three transfected cell lines.

### EGFRvIII-transfected cells are radiation sensitive

**Figure 2:**
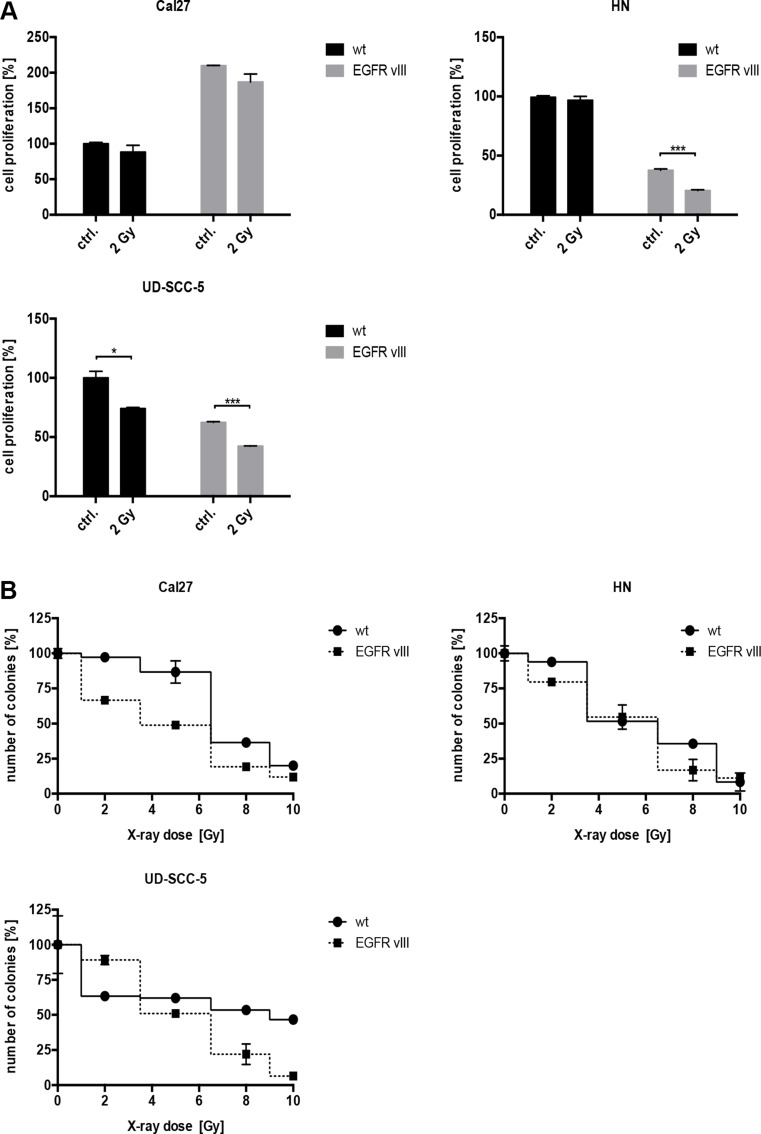
(**A**) The proliferation of the cells was detected via a crystal violet assay. Following radiation with 2 Gy, a significant decrease in the number of proliferating cells in the EGFRvIII-transfected cell lines HN, and UD-SCC-5 was observed. (**B**) Cell survival as measured by colony formation assay was decreased following irradiation with 2 to 10 Gy in all three cell lines transfected with EGFRvIII.

Evidence in the literature suggests that EGFRvIII-positive glioblastoma cells are more resistant to radiation [12], therefore, we conducted radiation experiments. After exposure to 2 Gy of radiation, a significant decrease in the number of proliferating cells was observed in the EGFRvIII-transfected cell lines, HN and UD-SCC-5 (Figure 2A). Additionally, cell survival measured by the colony formation assay was overall decreased following irradiation with 2 to 10 Gy in all three of the cell lines transfected with EGFRvIII (Figure 2B).

### HNSCC cells with and without EGFRvIII transfection exhibit a similar response to EGFR inhibition and radiation

**Figure 3:**
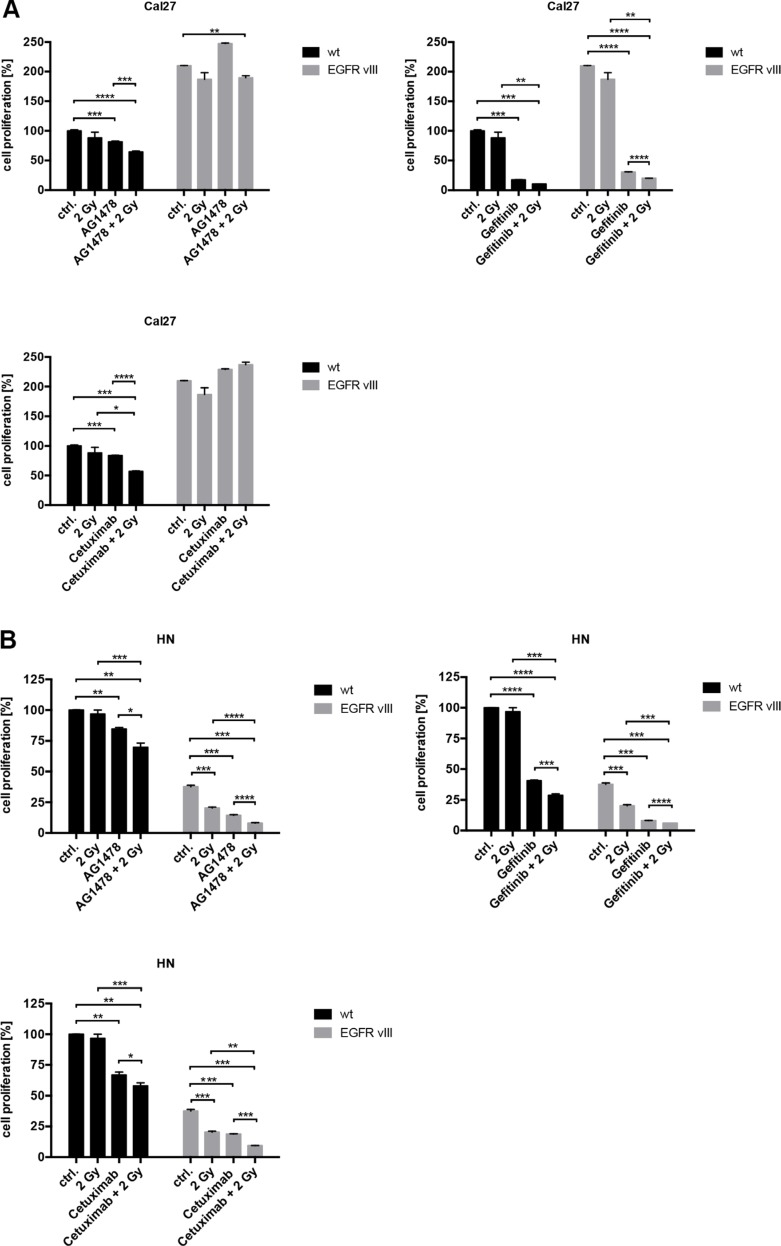

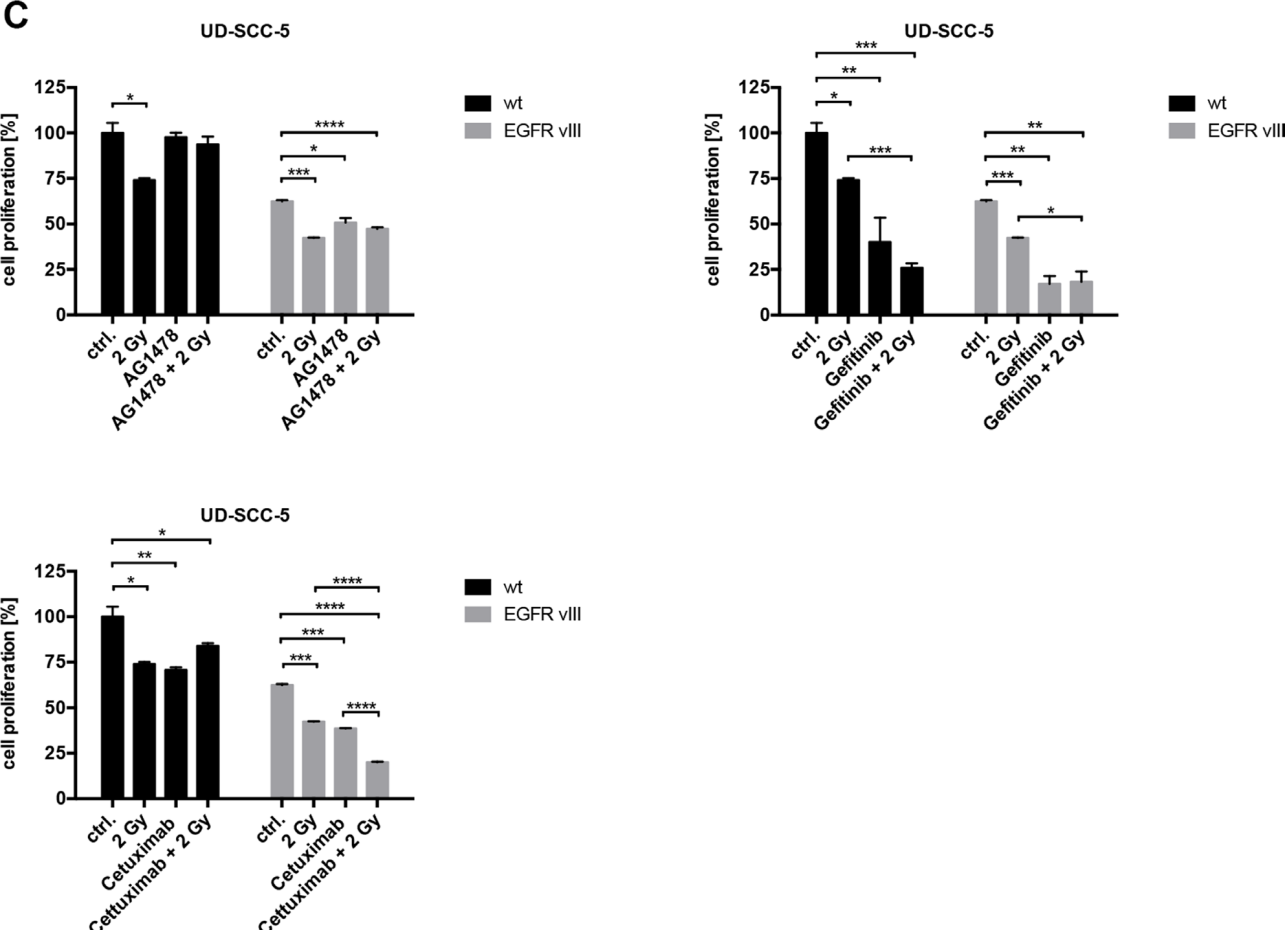
(**A**–**C**) The response to various EGFR inhibitors, radiation, and the combined treatment of EGFR inhibition and radiation was overall similar regarding the wild type and EGFRvIII-transfected cells as indicated by the crystal violet assays.

**Figure 4:**
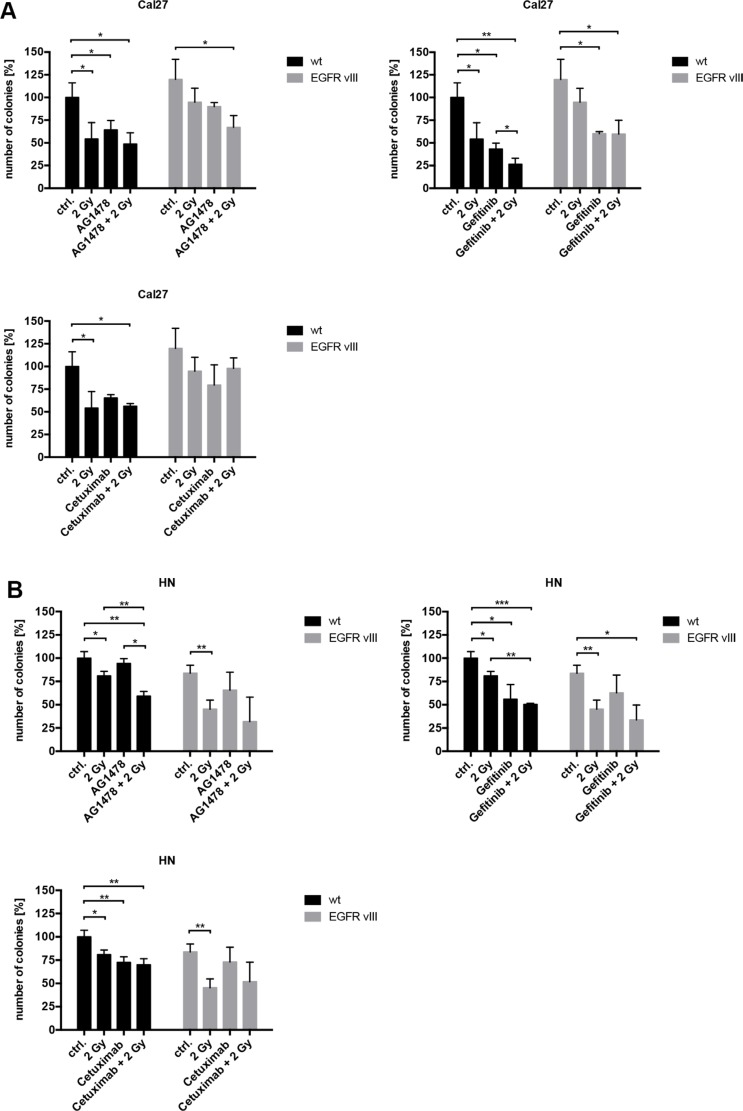

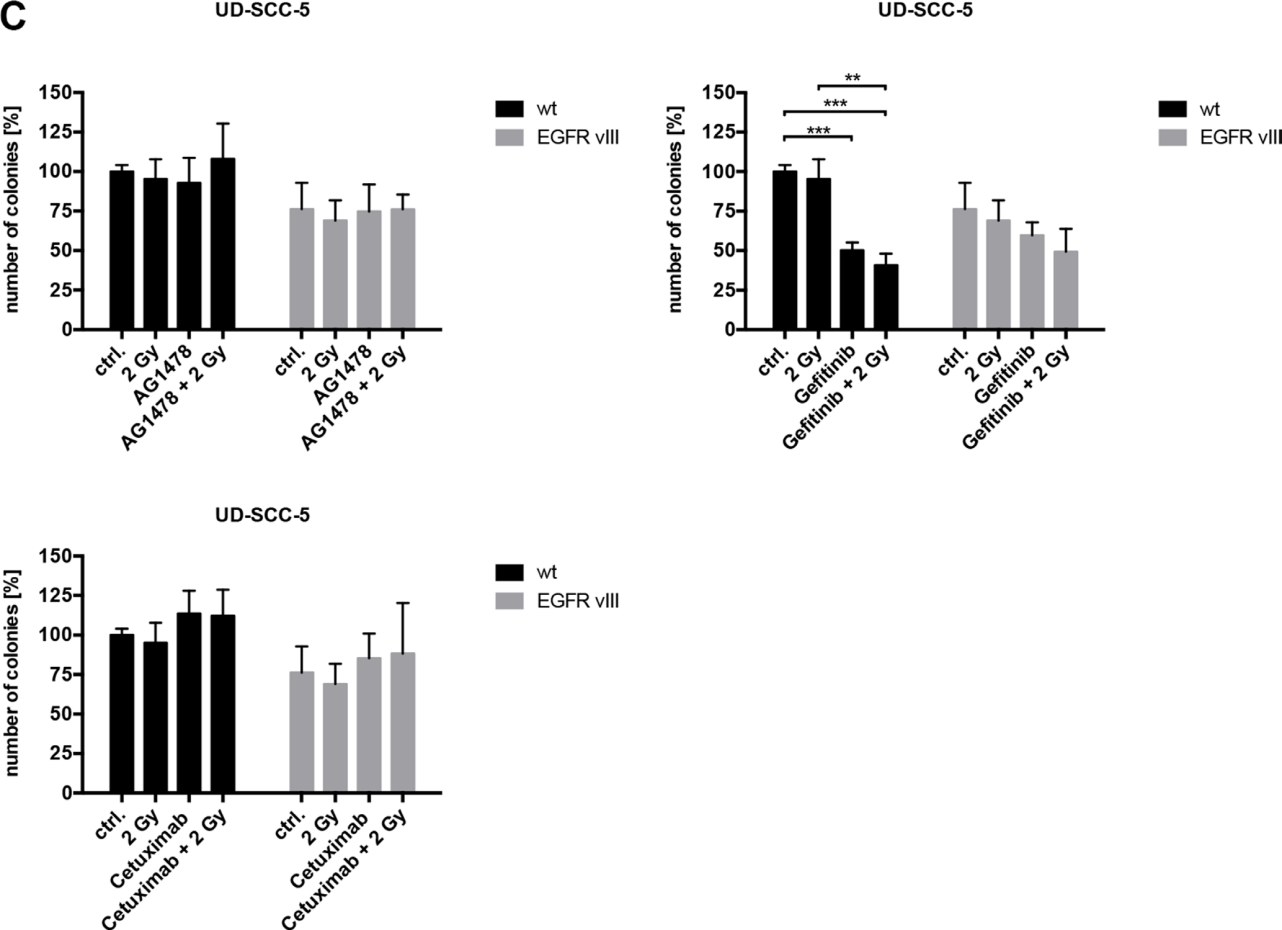
(**A**–**C**) Using colony formation assays, a decrease of cell survival was found in all three cell lines. The effect of EGFR inhibition and radiation was comparable between the wild type and EGFRvIII-transfected cells. Treatment with Gefitinib was the most effective.

To elicit the influence of EGFR inhibition, different inhibitors and antibodies were used (Supplementary Figure 1). In contrast to Cetuximab, a monoclonal EGFR antibody, Gefitinib and Tyrphostin AG1478 are both tyrosine kinase inhibitors (TKIs). These can inhibit intracellular tyrosine kinase activity and interact with both EGFR and EGFRvIII. The response to EGFR inhibition was found to be similar between the wild type and EGFRvIII-transfected cells. Only Cal27 EGFRvIII positive cells were more resistant to AG1478 and Cetuximab than Cal27 wild type cells in proliferation assay. Overall, similar results were found in cell proliferation and clonal survival (Figures 3 and 4). Treatment with Gefitinib was found most effective. Additionally, the combination of inhibition of the EGFR and radiation exhibited comparable response rates between the wild type and EGFRvIII-transfected cells.

### Radiation-induced cell migration is also exhibited by EGFRvIII positive cells

**Figure 5:**
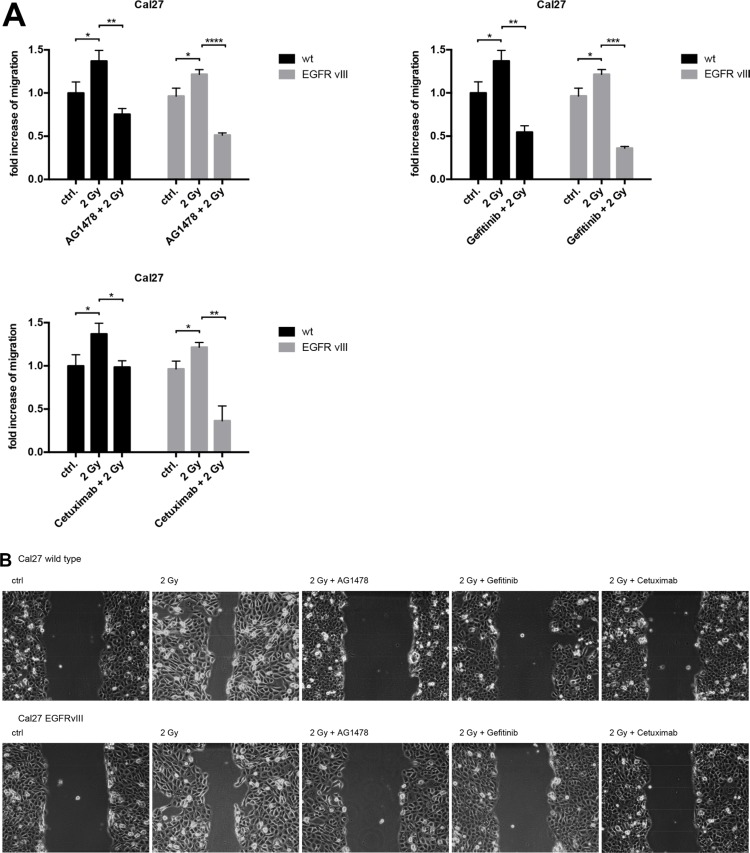
(**A**) Migration assays were conducted with the cell line Cal27. We observed radiation-induced migration in both cell groups (EGFRvIII positive and negative). Migration could be blocked significantly by inhibiting the EGF receptor with Tyrphostin AG1478, Gefitinib, and Cetuximab in addition to irradiation. (**B**) Histological pictures of the Cal27 migration assay illustrating the radiation-induced migration and its blocking in both cell groups with Tyrphostin AG1478, Gefitinib and Cetuximab in addition to irradiation.

To investigate the biological effect of radiation on EGFRvIII-transfected cells, migration assays were conducted in Cal27 cells. We found similar radiation-induced migration in both EGFRvIII-positive and negative cells. In both groups, migration was significantly blocked by the inhibition of the EGFR by Tyrphostin AG1478, Gefitinib, and Cetuximab in addition to radiation (Figure 5A and 5B).

### AKT and ERK phosphorylation in EGFRvIII-transfected cells despite EGF receptor blockage

**Figure 6:**
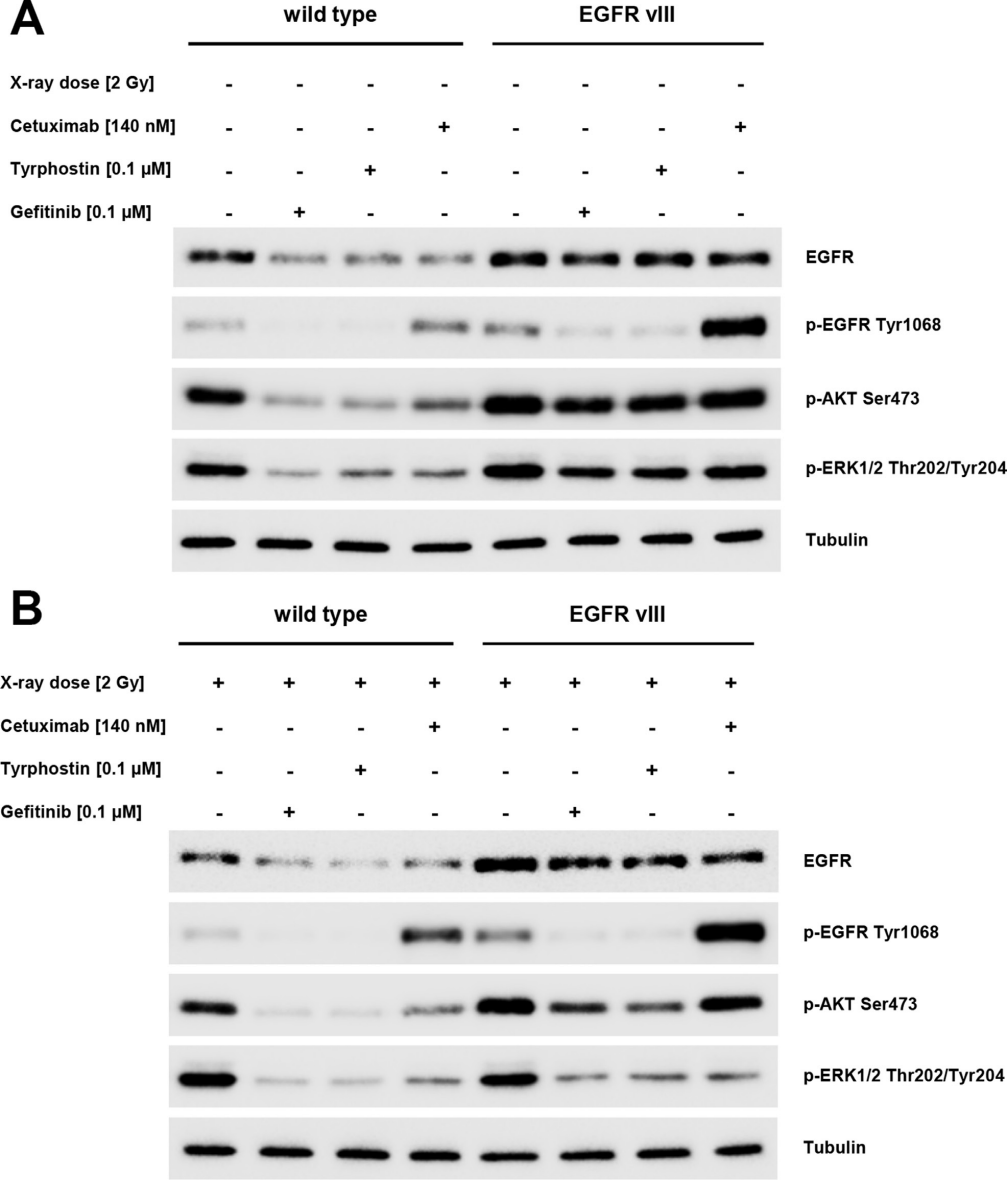
(**A** and **B**) Western blotting showed a reduction in the level of EGFR phosphorylation in EGFRvIII positive and negative cells by Gefitinib or Tyrphostin AG1478. Cetuximab led to an increased phosphorylation of the EGFR. The difference between the two groups was found regarding the EGFR downstream pathways AKT and ERK. We observed a phosphorylation of these two pathways despite blocking EGFR in EGFRvIII-positive cells. After radiation, the level of phosphorylation was reduced in these cells.

Using Western blotting, we observed a reduction in the levels of EGFR phosphorylation following a blockade using Gefitinib or Tyrphostin AG1478. In contrast, Cetuximab led to increased phosphorylation of the EGF receptor. These results were observed in both the wild type and EGFRvIII-transfected cells. A difference between these two groups was found in the EGFR downstream AKT and ERK pathways. We observed the phosphorylation of these two pathways despite blocking the EGFR in EGFRvIII positive cells. Following radiation, the phosphorylation decreased in these cells (Figure 6A and 6B).

### EGFRvIII can be detected in HNSCC and is not associated with prognosis

In contrast to other recent publications [10, 11] we were able to demonstrate EGFRvIII expression in HNSCC. Following RNA isolation and cDNA synthesis, we performed rtPCR with the five described primers in combination with gel electrophoresis for our first 113 cases. We found that the PCR results obtained with the five different primers was inconsistent. Only one sample was positively detected by all five primers. Therefore, sequencing was conducted with all putative EGFRvIII-positive cases and five cases of 113 were confirmed to be EGFRvIII-positive (5/113; 4.4%). Moreover, we conducted immunohistochemical staining using an EGFRvIII-specific antibody. However, only one sample was detected as EGFRvIII-positive. Remarkably, this sample was the same as that detected by all five primers and sequencing.

To eliminate the influence of long-term storage and fixation of EGFRvIII detection, we added 26 FFPE specimens obtained within the past two years (all embedded in 2014) and ten fresh-frozen specimens (all gathered in 2015). EGFRvIII was detected in 5 of 26 FFPE specimens (19.2%) and 3 of 10 fresh-frozen specimens (30%) using three of the five primers for PCR, gel electrophoresis, and sequencing. However, none of the detected cases were recognized by all three primers.

Taken together, only one out of 149 samples was detected to be positive by rtPCR and subsequent sequencing using all five primers and by immunohistochemical staining.

Additionally, we compared the overall survival of patients with EGFRvIII detected with at least one method (13/149) and the overall survival of patients without detection of EGFRvIII (136/149). No significant difference in overall survival was found (*p* = 0.618, Log Rank Test; Supplementary Figure 4).

## DISCUSSION

Squamous cell carcinoma of the head and neck is typically associated with an unfavorable prognosis, indicating that half of the affected patients cannot be cured [13]. Despite therapeutic advances, one challenging problem is the resistance mechanism of the EGF receptor against the monoclonal antibody, Cetuximab [14]. The EGFRvIII isoform appears to play a central role in the resistance mechanism in HNSCC [9].

The aim of this study was therefore to analyze the effectiveness of Cetuximab and the tyrosine kinase inhibitors (TKIs) Gefitinib, and Tyrphostin AG1478 with or without additional radiotherapy, on EGFR and EGFRvIII-expressing HNSCC cell lines. Furthermore, additional attention was paid to the frequency at which the EGFRvIII mutation was present in HNSCC patient tissue samples.

Between the wild type EGFR and EGFRvIII-expressing cells, similar response rates to the different inhibitors/antibodies were observed, and Gefitinib proved to be most effective. These results are similar to that achieved in studies of glioblastoma. Carrasco-Garcia et al. also showed the effective inhibition of the proliferation of glioblastoma cells by TKI, unlike that observed by Cetuximab [16]. Moreover, Baselga and Arteaga demonstrated in phase I trials that treatment of advanced NSCLC acquired tremendous success via an EGFR blockade by TKI [17]. The ineffective blockade of Cetuximab in both wild type and EGFRvIII cells was also observed in glioblastomas [16].

Additionally, the EGFRvIII transfected cell lines developed no radioresistance. Reasons for the resistance to ionizing radiation discussed in the literature include that radiation or Cetuximab-induced transport of the EGFR into the nucleus contributes to the development of resistance mechanisms [18]. Furthermore, Chaachouay et al. published that in radioresistant cell lines, autophagy induced by ionizing radiation might cause the development of such resistance. Based on this hypothesis, Schiefler et al. used the UD-SCC-5 cell line to demonstrate the effect of the induction of the radiation-dependent S1P-5 receptor. Among diverse functions, this receptor was found to be responsible for autophagy [19].

Cellular migration was also found to be similar between the wild type EGFR and EGFRvIII groups. We observed a reduction in migration after blocking the EGFR. The relevance of the EGFR pathway for radiation-induced migration has been previously described by our group [20].

The only distinction between the two groups was observed in protein expression and phosphorylation, as differences were observed in the EGFR downstream pathways, AKT and ERK. We observed the phosphorylation of these two pathways despite blocking the EGFR in EGFRvIII-expressing cells. Following radiation, the level of phosphorylation was reduced in these cells. The existing activity of the non-phosphorylated receptor in EGFRvIII justifies the biochemical function of the TKI. Specifically, the TKI does not lead to an internalization of the receptor and thus, leads to no reduction in protein expression [21]. For the AKT activation, a possible connection with the loss of PTEN is discussed in the literature. This activates EGFR-independent AKT, leading to resistance to EGFR inhibition [21].

The hyperphosphorylation of EGFR after Cetuximab treatment was unexpected and differed from the dephosphorylation following TKI therapy. Theoretically, after treatment with Cetuximab, a ligand-dependent blockade of the EGFR occurs. Thus, the receptor should be internalized and down-regulated, leading to the inhibition of downstream signaling cascade [21]. This paradoxical phosphorylation of EGFR by Cetuximab treatment has been described in the literature [22, 23].

In 1990, EGFRvIII was detected as a tumor-specific extracellular mutation of the EGF receptor for the first time in glioblastoma [24]. Moreover, this evidence is well established and reproducible. Having demonstrated the existence of this mutation in GBM, attempts were made to detect EGFRvIII in tumor tissues other than nervous system. However, the results of the existence of EGFRvIII in other malignancies are controversial. The use of only a single method for EGFRvIII detection and failure in the technical implementation have met with criticism. For example, Moscatello et al. published the detection of EGFRvIII in 75% of the investigated ovarian cancer cases using only Western Blotting [25]. However, these results could not be reproduced by other research groups that applied reliable and multiple detection methods [6]. In 2002, an American study demonstrated an incidence of 67.8% EGFRvIII mRNA transcripts in primary human breast cancer by rtPCR [26]. In turn, Wildstrand et al. found only 27% of breast cancer samples to be EGFRvIII-positive [6]. Similar discrepancies have been observed in colorectal cancer and non-small cell lung cancer tissues [6]. In tumors of the head and neck region, the EGFRvIII expression rates even vary between 0% and 80% [9, 11]. In 2014, Melchers et al. investigated the existence of EGFRvIII in head and neck tumors. They used a dual detection method via immunohistochemical staining and rtPCR. Due to the different results obtained by the two detection methods, the existence of EGFRvIII was denied [11]. Similarly, in the present study, all five primers obtained from various publications provided very different results. The differing rate of detection could also in part be due to unspecific off target amplification by primers. In addition, the results were also affected by the age of the tissue. Ultimately, only one case was shown to be EGFRvIII-positive using all the detection methods.

## MATERIALS AND METHODS

### Cell culture

The Cal27 and HN cell lines were obtained from DSMZ (Braunschweig, Germany) and the UD-SCC-5 cell lines were obtained from the University of Düsseldorf (Department of Otorhinolaryngology, Düsseldorf, Germany). All cell lines presented a moderate expression of EGFR by Western blotting. The cells were cultured in Dulbecco's Modified Eagle Medium (DMEM) (Invitrogen, Darmstadt, Germany) containing 10% fetal bovine serum (FBS) (Biochrom, Berlin, Germany), 2 mM glutamine, 100 μg/mL streptomycin, and 100 U/mL penicillin (Biochrom, Berlin, Germany), maintained at 37°C in an atmosphere of 5% CO_2_, and grown to 70%–90% confluence.

### Transfection

All the three cell lines were transfected with the pLEARN-EGFRvIII plasmid. The plasmid was kindly provided by Prof. Keller (III. Medizinische Klinik, Klinikum rechts der Isar, Technical University of Munich, Germany).

### Inhibitors

The mouse-human chimeric EGFR-inhibitory antibody, Cetuximab, was purchased from Merck (Merck, Darmstadt, Germany). Tyrphostin AG1478 (Sigma-Aldrich Chemie, Steinheim, Germany) and Gefitinib (Selleckchem, Houston, Texas, USA) were also applied.

### X-ray irradiation

Irradiation was performed at the Department of Radiotherapy (Klinik für Radioonkologie und Strahlentherapie, Klinikum rechts des Isar, Technical University of Munich, Germany). The cells were X-ray irradiated at room temperature, 24 h after seeding using a Gulmay CP-2225 Medical X-ray source operated at 70 kV and a dose rate of approximately 1 Gy/min. The sham-treated group (0 Gy, control) was subjected to the same protocol as the exposed cells.

### Analysis of cell proliferation

The binding of crystal violet to cellular DNA was used to assess cell proliferation via ELISA. Cells were seeded at a density of 5 × 10^3^ cells per well in six-well plates 24 h before treatment. Ten days after the treatment with the inhibitors or X-rays, the culture medium was aspirated and 500 μL of 4% formaldehyde was added to each well for 30 minutes. After washing with 0.1% Triton-X100/PBS and H_2_O, crystal violet (0.04%) was added to the fixed cells and allowed to act for 30 minutes. Finally, SDS (1%) was added, and the optical density was measured at 590 nm using an ELISA reader after 1 h.

### Colony formation assay

Cell survival after treatment with inhibitors or X-rays was assessed using a colony formation assay (CFA). The cells were seeded in six-well plates (5 × 10^2^ cells per well) and allowed to adhere overnight at 37°C. The following day, the cells were treated and incubated at 37°C for 10 days. The cell colonies were then formalin fixed (4% formaldehyde) and visualized by Crystal Violet (0.04%) staining (Sigma-Aldrich, Steinheim, Germany). The colonies were counted after washing off the dye. Colony numbers were depicted as the percentage of colonies from untreated cells using GraphPad Prism.

### Wound healing assay (migration assay)

Cell migration was assessed by the wound-healing assay (WHA). The cells were seeded in six-well plates (7.5 × 10^5^ cells per well), and a scratch was drawn into the cell layer after 24 h of incubation. The cells were then pretreated with the inhibitors or X-ray irradiated. Pictures of the scratch were obtained immediately and 12 h after the treatment. The number of pixels covered with cells were evaluated using Photoshop, and the magnitude of the points of measurement was compared using the following formula:

(T2-T1)/5038848 × 100

(T1 = 5038848 – the number of pixels at the start of measurement)

(T2 = 5038848 – the number of pixels at 12 h)

(5038848 = the total number of pixels)

### Western blotting

For protein expression analysis, the cells were lysed in a lysis buffer (New England Biolabs, Frankfurt, Germany) supplemented with 1 mM PMSF (Roth, Karlsruhe, Germany). Protein concentrations of the lysates were quantified using Bradford assay to ensure equal amounts of protein were loaded per lane in the SDS-PAGE. Fifteen microgram protein was separated by SDS-PAGE and transferred to Immobilon PVDF membranes (Millipore, Schwalbach, Germany). Blocking of unspecific binding sites was performed using 5% (w/v) non-fat dry milk in TBST. The membranes were incubated with primary antibodies diluted in TBST for 12 to 14 h at 4°C. HRP-conjugated immunoglobulins (diluted 1:5000 in 5% non-fat dry milk/TBST) served as detection antibodies and were incubated for 1 h at room temperature. Immunoreaction was visualized using the Pierce ECL Western Blotting Substrate (Thermo Scientific) and exposure to high-performance chemiluminescence film (GE Healthcare, Freiburg, Germany).

Primary antibodies against the following antigens were used: p-EGFR Tyr1068 (Rabbit) (New England Biolabs) (1:2500); EGFR (Rabbit) (Santa Cruz Biotechnology, Dallas, Texas) (1:2500); L8A4 EGFRvIII specific antibody (kindly provided by Dr. Darell Bigner, Duke University Medical Center, Durham, USA) (1:500); p-Akt Ser473 (Rabbit) (New England Biolabs) (1:500); p-Erk1/2 Thr202/Tyr204 (Rabbit) (New England Biolabs) (1:1000); and tubulin (Mouse) (Sigma-Aldrich Chemie, Steinheim, Germany) (1:5000).

### Patient tissue samples

Tumor tissue samples of 149 head and neck squamous cell carcinoma (HNSCC) patients were used. All the patients were treated in the Department of Otorhinolaryngology at the Klinikum rechts der Isar, Technical University Munich, Germany.

The first subgroup consisted of 113 specimens, which were all formalin fixed and paraffin embedded (FFPE). The FFPE tissue samples of this group were older than two years (mean: 11 years old, range: 2 to 21 years). Therefore, an additional subgroup of 26 FFPE specimens younger than two years (all embedded in 2014) and ten fresh-frozen specimens (all gathered in 2015) were added to examine the influence of long-term storage and fixation on EGFRvIII detection.

The study was approved by the independent ethics committee of the Technical University of Munich (project number 1420/05).

### Clinical data

Clinical data was retrieved from the local clinical information system and filed medical records were obtained by hand searching. Detailed patient characteristics and histomorphological features are provided in Table 1.

**Table 1:** Clinical characteristics of the patients included in the study

Sex	Male	126	84.6%
	Female	23	15.4%
Tumor localization	Oral cavity	20	13.4%
	Oropharynx	54	36.2%
	Hypopharynx	13	8.7%
	Larynx	26	17.4%
	Nasopharynx	0	0.0%
	Sinonasal	36	24.2%
Tumor stage	T1	55	36.9%
	T2	46	30.9%
	T3	27	18.1%
	T4	17	11.4%
	Unknown	4	2.7%
Nodal stage	N0	74	49.7%
	N1	15	10.1%
	N2	50	33.6%
	N3	4	2.7%
	Unknown	6	4.0%
Distant metastasis	M0	131	87.9%
	M1	3	2.0%
	Unknown	15	10.1%
Histologic grade	G1	8	5.4%
	G2	82	55.0%
	G3	55	36.9%
	G4	3	2.0%
	Unknown	1	0.7%

### Immunohistochemistry

Immunohistochemical staining was conducted with the FFPE tissue derived from 113 HNSCC samples. The L8A4 EGFRvIII specific antibody (kindly provided by Dr. Darell Bigner, Duke University Medical Center, Durham, USA) that binds EGFRvIII at the specific protein sequence generated by the junction of exon 1 and exon 8 of the wild type EGFR was used.

Fresh 2 μm sections from FFPE blocks were transferred onto glass slides and stained by hand or machine-stained with Leica Bond-Max (Leica Biosystems Nussloch GmbH, Nussloch, Germany). The slides were deparaffined and rehydrated. After a peroxide block, the antigen retrieval was performed by heating in EDTA buffer. The slides were cooled down and incubated with the L8A4 EGFRvIII antibody. The reaction was developed with the ZytoChem-Plus Horseradish Peroxidase Polymer-Kit (Zytomed Systems GmbH, Berlin, Germany) according to the manufacturer's protocol respectively with Leica's own machine optimized solutions. DAB was used as the reaction indicator. After counterstaining with hematoxylin, the slides were dehydrated in ascending concentrations of ethanol and mounted. EGFRvIII positive glioblastoma tissue served as a positive control (shown in Supplementary Figure 2).

### RNA extraction and cDNA synthesis

For real-time reverse transcriptase PCR (rtPCR) analysis, the RNA was isolated from all 149 HNSCC cases. For RNA isolation, 139 FFPE HNSCC specimens were dewaxed and 10 fresh-frozen HNSCC specimens were homogenized in Proteinase K buffer (50 mM Tris and 1 mM EDTA diluted in water). After dewaxing respectively homogenization the samples were digested with 80 μL Proteinase K (Roche Diagnostics GmbH, Unterhaching, Germany), 200 μL Proteinase K buffer plus Tween-20 (Tween 20 25% one part added to fifty parts of Proteinase K buffer) and 32 μL 10% sodiumdodecylsulfate overnight at 55°C. The next day, another 10 μL Proteinase K was added for further incubation overnight. Following digestion, the RNA isolation was continued with the InviTrap Spin Universal RNA Mini Kit (Stratec, Birkenfeld, Germany) according to the manufacturer's protocol. Finally, the RNA concentration of the samples was measured with the NanoDrop 1000 system (PEQLAB, Erlangen, Germany) and the samples were processed immediately or stored at −20°C until further processing. Only RNA probes with an Absorbance_260/280_ between 1.5 and 2.2 and a minimal RNA concentration of 5 ng/μL were used. The cDNA was then synthesized using Maxima^®^ reverse transcriptase (Thermo Fisher Scientific, Waltham, USA) according to the manufacturer's protocol.

### Real-time reverse transcriptase PCR, gel electrophoresis, and sequencing

All 149 isolated HNSCC samples were analyzed by rtPCR. For the establishment of primers, EGFRvIII positive glioblastoma tissue was used. In addition, EGFRvIII plasmid and EGFRvIII transfected cell lines previously established in our laboratory were used. Five already published EGFRvIII primers were established. All five primers flank the EGFRvIII-characteristic deletion of exon 2–7 and lead to short PCR products if EGFRvIII was detectable. In addition, a much longer PCR product could be generated due to the wtEGFR cDNA sequence. The primers used are listed in Table 2.

**Table 2:** Primers used in this study

	Primer sequence: forward	Primer sequence: reverse	PCR product [bp]	Publication
Primer 1	5′-GGCTCTGGAGGAAAAGAAAG-3′	5′-TCCTCCATCTCATAGCTGTCG-3′	90	Yoshimoto et al.[27]
Primer 2	5′-TGCTGGCTGCGCTCTGC-3′	5′-CACAGGCTCGGACGCAC-3′	92	Melchers et al.[11]
Primer 3	5′-GGAGCAGCGATGCGACCCTC-3′	5′-ACACTTGCGGACGCCGTCTT-3′	187	Boeckx et al.[28]
Primer 4	5′-ATGCGACCCTCCGGGACG-3′	5′-ATTCCGTTACACACTTTGCGGC-3′	236	Sok et al.[8].
Primer 5	5′-GAGCTCTTCGGGGAGCAG-3′	5′-GTGATCTGTCACCACATAATTACCTTTCT-3′	131	Yoshimoto et al.[27]

The existence of wild type EGFR was demonstrated with a pair of primers located in exon 1 and 2. The sequences 5′-TGCTGGCTGCGCTCTGC-3′ (forward, exon 1) and 5′-GAACATCCTCTGGAGGCTGAGA-3′ (reverse, exon 2) published by Melchers et al. lead to 125 bp amplicons [11]. Since EGFRvIII lacks exon 2–7, EGFRvIII was not amplified by these primers.

The specific annealing temperatures for the different primers were tested using gradient PCRs with different repeat cycle numbers followed by gel electrophoresis (annealing temperatures: Primer 1: 57.0°C, Primer 2: 64.5°C, Primer 3: 64.5°C, Primer 4: 61.4°C, and Primer 5: 64.5°C). All primers pairs were established using cDNA from positive GBM tissue and with EGFRvIII plasmid cDNA (Supplementary Figure 3).

The PCR mix for each probe contained 12.5 μL KAPA-SYBR Fast Universal (PeqLab, Erlangen, Germany), 0.5 μL (0.8 pmol) forward primer, 0.5 μL (0.8 pmol) reverse primer, 2 μL templates, and 9.5 μL water. The PCR program commenced by heating the samples at 95°C for 15 minutes. Next, there were 45 cycles at 95°C for 30 seconds, the specific annealing temperature for 30 seconds, and 30 seconds at 72°C.

The PCR products were then analyzed by gel electrophoresis. Gel electrophoresis was performed using 8 μL of DNA and RNA Dye peqGREEN (PeqLab, Erlangen, Germany) added per 100 mL of melted 2% TBE agarose solution. A volume of 10 μL PCR product mixed with 1.8 μL Blue Loading Buffer (PeqLab, Erlangen, Germany) was loaded on the gel.

All putative EGFRvIII positive cases were sequenced. Sequencing was performed through MWG Eurofins (Ebersberg, Germany) with cycle sequencing technology (dideoxy chain termination/cycle sequencing) on an ABI 3730XL sequencing machine.

### Statistical analyses

For interpretation of the immunohistochemical analysis, PCR, and sequencing data, statistical analyses were performed using SPSS software (version 23, IBM). Statistical analyses of the results from the *in vitro* experiments were performed using the Prism Graph Pad 6.0 software. A significance level of 5% was used as the threshold for all statistical tests.

## CONCLUSIONS

EGFRvIII can be detected in HNSCC; however, the low prevalence casts doubt on the clinical relevance of EGFRvIII. Additionally, further questioning the relevance of EGFRvIII in HNSCC, the EGFRvIII transfected cell lines did not show increased resistance to irradiation or EGFR targeted drugs compared to wild type cell lines.

The fact that the primers used yielded variable results could explain the inconsistent data in the literature. Therefore, future examinations should be conducted with more than one primer, in addition to sequencing and immunohistochemistry to achieve comparable results.

## SUPPLEMENTARY MATERIALS FIGURES


