# Supplementary material for: Epidermal growth factor receptor variant III in head and neck squamous cell carcinoma is not relevant for targeted therapy and irradiation
Source: Oncotarget. 2017 Mar 6;8(20):32668–82. doi: 10.18632/oncotarget.15949 (PMC5464818; doi:10.18632/oncotarget.15949)
Supplement: Supplementary file 1 [file oncotarget-08-32668-s001.pdf]

Epidermal growth factor receptor variant III in head and neck squamous cell carcinoma is not relevant for targeted therapy and irradiation

Supplementary Materials

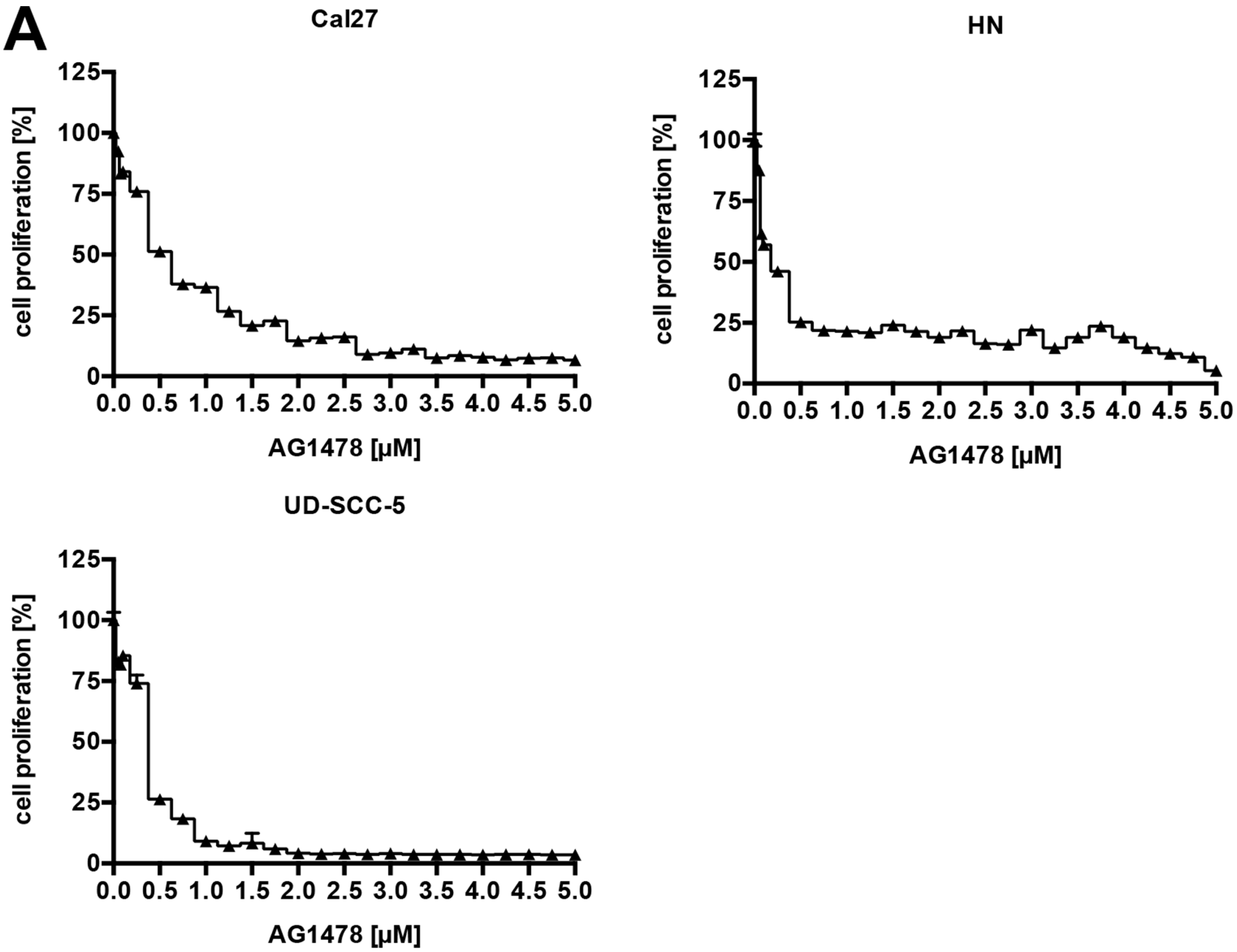

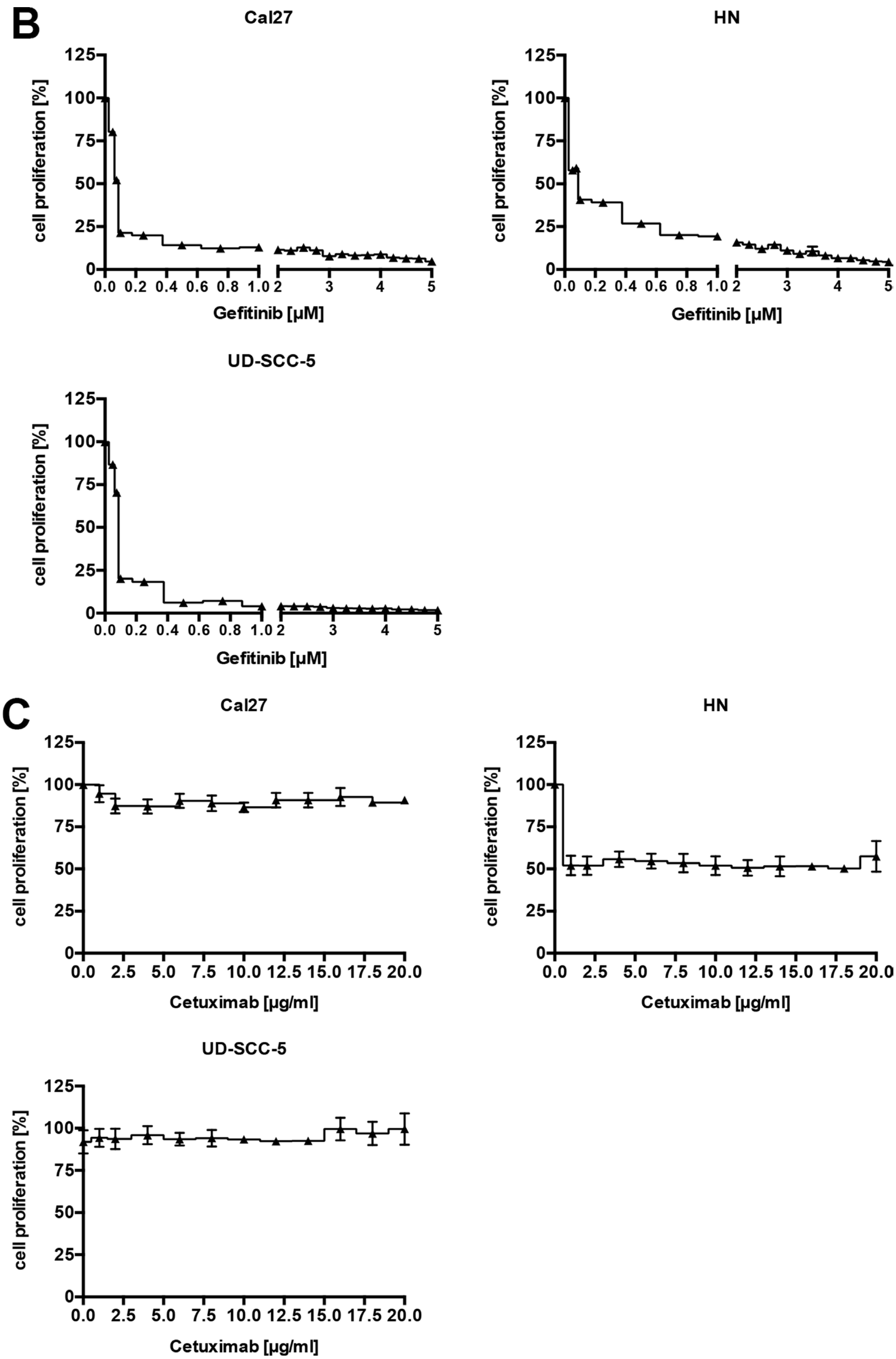

Supplementary Figure 1: Kinetics for the antibodies and inhibitors used in this study are presented. the tyrosine kinase inhibitors, Tyrphostin AG1478 (**A**) and Gefitinib (**B**) and the antibody against the EGF receptor Cetuximab (**C**).

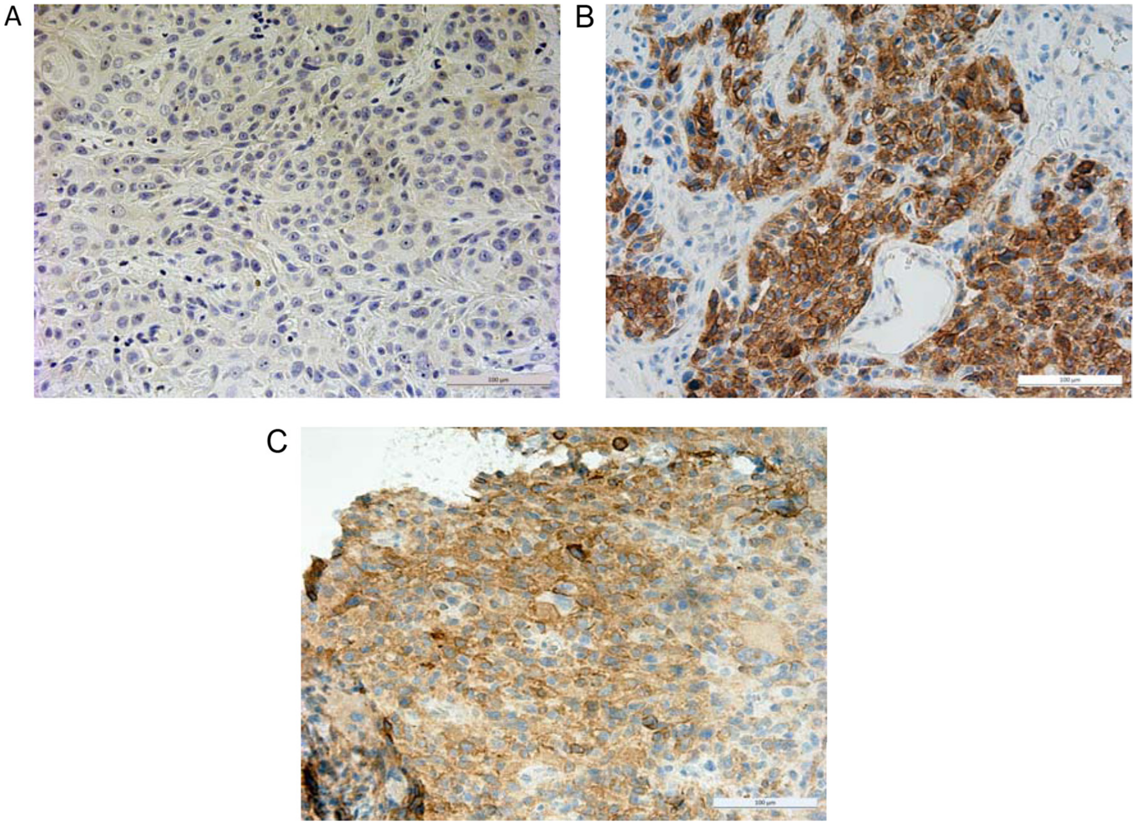

Supplementary Figure 2: Immunohistochemical staining of EGFRvIII. EGVRvIII negative HNSCC tissue sample (**A**), EGFRvIII positive HNSCC tissue sample (**B**), and EGFRvIII positive glioblastoma tissue sample (**C**).

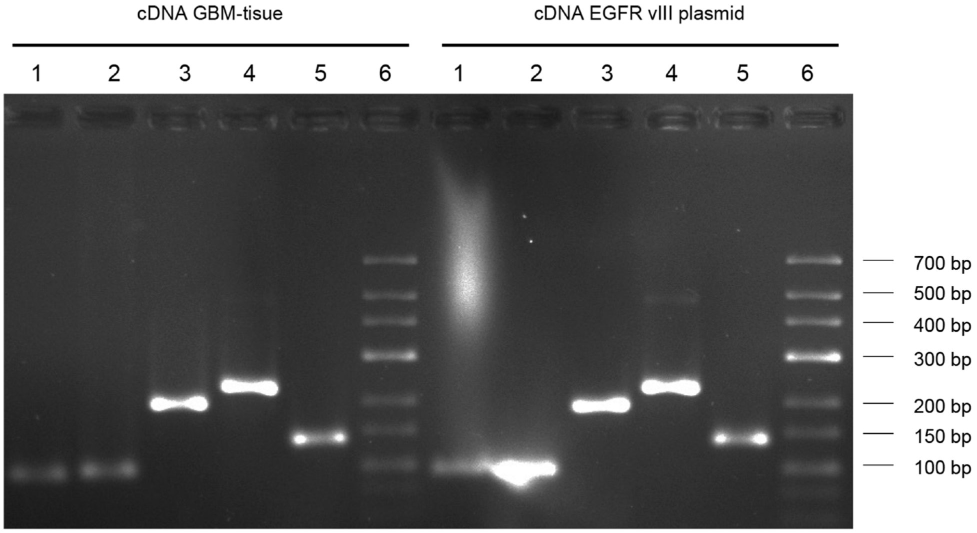

lane	treatment
1	Primer 1
2	Primer 2
3	Primer 3
4	Primer 5
5	Primer 4
6	Ultra Low Range II

Supplementary Figure 3: Gel electrophoresis: rtPCR amplification products of EGFRvIII positive glioblastoma tissue and EGFRvIII plasmid cDNA using the five different primers (DNA marker: PeqLab Ultra Low Range II).

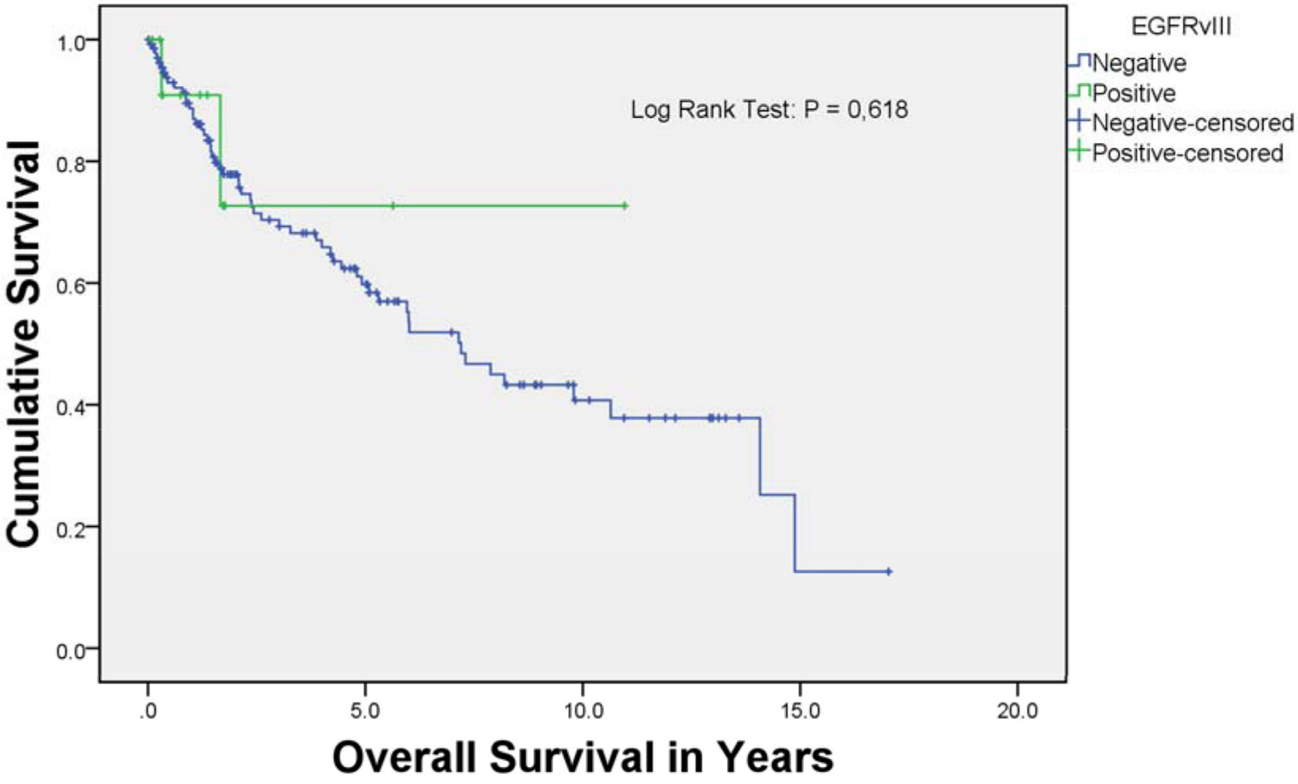

Supplementary Figure 4: Survival analysis of EGFRvIII negative patients versus EGFRvIII positive (one case) and EGFRvIII positive patients only detected by one method.
